# Draft Genome Sequence of *Rhodococcus erythropolis* DN1, a Crude Oil Biodegrader

**DOI:** 10.1128/genomeA.00846-13

**Published:** 2013-10-17

**Authors:** Alexander Shevtsov, Pavel Tarlykov, Elena Zholdybayeva, Dauren Momynkulov, Ainur Sarsenova, Nazira Moldagulova, Kuvat Momynaliev

**Affiliations:** National Center for Biotechnology, Astana, Kazakhstan

## Abstract

We report the 6,548-Mb genome sequence of *Rhodococcus erythropolis* strain DN1, isolated from the oil-contaminated soil in the Karagandy region of Kazakhstan. The draft genome sequence of strain DN1 might provide new insights into the genetic mechanisms of crude oil biodegradation.

## GENOME ANNOUNCEMENT

*Rhodococcus erythropolis* is a member of the aerobic Gram-positive bacteria, famous for their ability to catabolize a wide range of organic compounds, including alkanes, cycloalkanes, and various aromatic hydrocarbons (1–5). *R. erythropolis* strain DN1 was isolated from oil-contaminated soil in the Karagandy region of central Kazakhstan. Cultivation experiments with 3% crude oil solution resulted in the solution being almost entirely (92.7%) degraded by DN1 after 30 days of incubation. In addition, DN1 was able to degrade crude oil in the temperature range of 5°С to 37°С and in high NaCl concentrations (up to 6.5%).

The whole-genome shotgun (WGS) sequence data were retrieved with Ion Torrent sequencing technology that gave approximately 130-fold coverage. A total of 78 contigs, with an N_50_ length of 224,624 bp and a G+C content of 62.4%, were assembled using Newbler software version 2.3 (454 Life Sciences). The genome was annotated using the NCBI Prokaryotic Genomes Automatic Annotation Pipeline, released in 2013. The draft whole-genome sequence of strain DN1 has 6,548,397 bases that constitute 5,979 predicted coding sequences (CDSs) and 3 rRNA and 54 tRNA genes. The open reading frame (ORF) annotation by Rapid Annotations using Subsystems Technology (RAST) (version 4.0) (6) has indicated that many proteins are potentially involved in the metabolism of different organic compounds, including amino acids and derivatives (588 ORFs), carbohydrates (520 ORFs), cofactors, vitamins, prosthetic groups, and pigments (453 ORFs), and fatty acids, lipids, and isoprenoids (386 ORFs); protein metabolism (237 ORFs); DNA and RNA metabolism (219 ORFs); and the metabolism of aromatic hydrocarbons (61 ORFs) and other compounds. In addition, an analysis of the DN1 CDSs revealed that many genes, including those for aliphatic and aromatic dioxygenases, are involved in the biodegradation of xenobiotics. It is reasonable to suggest that strain DN1 will be useful in the bioremediation of regular oil-contaminated soils and oil-contaminated soils with high salt concentration.

### Nucleotide sequence accession numbers.

The whole-genome shotgun project has been deposited at DDBJ/EMBL/GenBank under the accession no. AUZK00000000. The version described in this paper is the first version, AUZK01000000.
